# Autosomal Recessive Polycystic Kidney Disease

**Published:** 2014-04-01

**Authors:** Sajad Ahmad Salati

**Affiliations:** Department of Surgery, College of Medicine, Qassim University, Saudi Arabia.

**Dear Sir**

A 25-year-old pregnant female reported at 28 weeks of gestation with non specific abdominal pain. On evaluation, the ultra-sonogram revealed a single live fetus in breech presentation. Placenta was anterior and high and there was severe oligohydramnios. Both fetal kidneys were enlarged in size (occupying most of the abdominal cavity) and homogenously hyperechoic & studded with numerous variable sized cysts. Besides, cisterna magna was also prominent. There was no other significant past medical or surgical history and she had delivered one healthy male child before three years. Both spouses were normal on clinical evaluation and there was no family history of renal diseases on the either side. Ultrasonogram ruled out any features of renal diseases in both spouses and the first child. The diagnosis of autosomal recessive polycystic kidney disease (ARPKD) was made on the basis of fetal imaging and absence of any family history of renal disorders. The pregnancy delivered vaginally at 32 weeks of gestation. The baby had severe respiratory distress and died within the first few hours of life. No pathological post-mortem or DNA analysis was possible due to logistic issues.

ARPKD has an incidence of 1 in 6,000 to 55,000 infants.[1] The causative factor is the mutations in the PKHD1 (chromosomal locus 6p12.2).[2] The disease has a wide clinical variation from stillbirth and neonatal demise to survival into adulthood; the carrier frequency for a PKHD1 mutation is estimated to be about 1:70 in general population. The majority of cases present in the neonatal period though the diagnosis may be suspected on prenatal ultrasound. Severely affected foetuses display a Potter-like facies [1] oligohydramnios phenotype with lethal pulmonary hypoplasia and massively enlarged echogenic kidneys, as in the above presented case. About 23%-30% of affected infants die in the neonatal period as a result of respiratory insufficiency or superimposed pulmonary infections. The survivors develop features of renal dysfunction and more than 50% of affected cases progress to end-stage renal disease (ESRD). With the availability of dialysis and kidney and/or liver transplantation, their ten-year survival in has improved to 82%. Rarely the disease may manifest in adolescence or adulthood with features of hepatic dysfunction including portal hypertension, esophageal varices, and hypersplenism. High resolution ultrasonography is the primary radiographic modality for the evaluation of autosomal recessive polycystic kidney disease (ARPKD), especially during the perinatal and neonatal periods.[3] The characteristic findings in ultrasonogram are enlarged, homogenously hyper-echogenic kidneys with the absence of cortico-medullary differentiation.[4] In older children, CT and MRI have a significant role in evaluation of the liver disease.[5]

**Figure:**
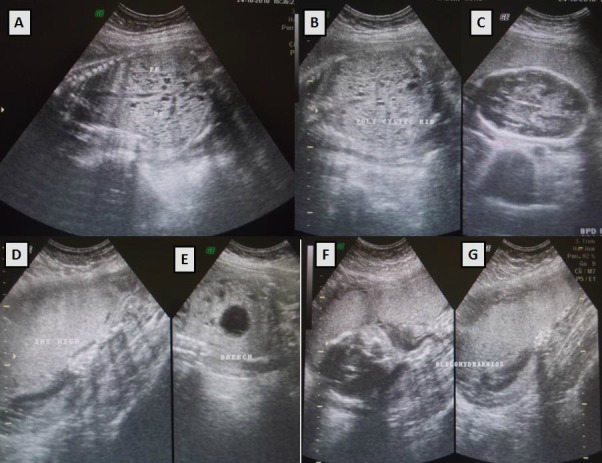
Figure 1: High resolution fetal ultrasonogram depicting (A , B, C) Hyperechoic and enlarged kidneys characteristic of in autosomal recessive polycystic kidney disease; (D) High, anterior placenta; (E) Breech presentation; (F, G)Oligohydramnios

## Footnotes

**Source of Support:** Nil

**Conflict of Interest:** None

